# Cancer-Related Characteristics Associated With Invasive Mechanical Ventilation or In-Hospital Mortality in Patients With COVID-19 Admitted to ICU: A Cohort Multicenter Study

**DOI:** 10.3389/fonc.2021.746431

**Published:** 2021-11-30

**Authors:** Pedro Caruso, Renato Scarsi Testa, Isabel Cristina Lima Freitas, Ana Paula Agnolon Praça, Valdelis Novis Okamoto, Pauliane Vieira Santana, Ramon Teixeira Costa, Alexandre Melo Kawasaki, Renata Rego Lins Fumis, Wilber Antonio Pino Illanes, Eduardo Leite Vieira Costa, Thais Dias Midega, Thiago Domingos Correa, Fabrício Rodrigo Torres de Carvalho, Juliana Carvalho Ferreira

**Affiliations:** ^1^ Intensive Care Unit, AC Camargo Cancer Center, Sao Paulo, Brazil; ^2^ Divisao de Pneumologia, Instituto do Coracao (InCor), Hospital das Clinicas HCFMUSP, Faculdade de Medicina, Universidade de Sao Paulo, Sao Paulo, Brazil; ^3^ Hospital Sírio-Libanês, Research and Education Institute, Sao Paulo, Brazil; ^4^ Department of Critical Care Medicine, Hospital Israelita Albert Einstein, Sao Paulo, Brazil

**Keywords:** cancer, COVID-19, intensive care unit, neoplasms, hospital mortality, respiration, artificial

## Abstract

**Background:**

Coexistence of cancer and COVID-19 is associated with worse outcomes. However, the studies on cancer-related characteristics associated with worse COVID-19 outcomes have shown controversial results. The objective of the study was to evaluate cancer-related characteristics associated with invasive mechanical ventilation use or in-hospital mortality in patients with COVID-19 admitted to intensive care unit (ICU).

**Methods:**

We designed a cohort multicenter study including adults with active cancer admitted to ICU due to COVID-19. Seven cancer-related characteristics (cancer status, type of cancer, metastasis occurrence, recent chemotherapy, recent immunotherapy, lung tumor, and performance status) were introduced in a multilevel logistic regression model as first-level variables and hospital was introduced as second-level variable (random effect). Confounders were identified using directed acyclic graphs.

**Results:**

We included 274 patients. Required to undergo invasive mechanical ventilation were 176 patients (64.2%) and none of the cancer-related characteristics were associated with mechanical ventilation use. Approximately 155 patients died in hospital (56.6%) and poor performance status, measured with the Eastern Cooperative Oncology Group (ECOG) score was associated with increased in-hospital mortality, with odds ratio = 3.54 (1.60–7.88, 95% CI) for ECOG =2 and odds ratio = 3.40 (1.60–7.22, 95% CI) for ECOG = 3 to 4. Cancer status, cancer type, metastatic tumor, lung cancer, and recent chemotherapy or immunotherapy were not associated with in-hospital mortality.

**Conclusions:**

In patients with active cancer and COVID-19 admitted to ICU, poor performance status was associated with in-hospital mortality but not with mechanical ventilation use. Cancer status, cancer type, metastatic tumor, lung cancer, and recent chemotherapy or immunotherapy were not associated with invasive mechanical ventilation use or in-hospital mortality.

## Introduction

It has been shown that patients with cancer are more susceptible to the disease caused by the new SARS-CoV-2 virus (COVID-19) (1, 2) and that the coexistence of cancer and COVID-19 is associated with worse outcomes, such as hospitalization, invasive mechanical ventilation (MV) use, intensive care unit (ICU) admission, and mortality (1–5). However, the studies that evaluated the cancer-related characteristics associated with worse COVID-19 outcomes have shown controversial results.

In some studies, recent chemotherapy (6–10), recent immunotherapy (11), type of cancer (3, 6, 9–12), worse performed status (10, 13, 14), lung as the primary site of the solid tumor (1, 6, 12), metastatic tumor (3), and the cancer status (8, 10, 13, 15, 16) were associated with worse COVID-19 outcomes, while other studies showed that recent chemotherapy (11, 12, 14, 17–20), recent immunotherapy (10, 19), type of cancer (15, 21), worse performed status (12, 20), lung cancer (3, 14, 16, 17), metastatic tumor (11, 12, 14, 18, 19), and the cancer status (8, 12, 19), were not associated with worse outcomes (**Supplementary Table 1**).

One plausible explanation for the controversial results is the heterogeneity of the evaluated population. Most studies included outpatients and inpatients in different proportions, including patients with different disease severity, ranging from asymptomatic (20) to hospitalized patients (3, 7, 8). However, no study evaluated only critically ill patients with active cancer admitted to ICU due to COVID-19, that are the patients with the higher probability of MV use and death.

Better understanding of the cancer-related characteristics associated with worse outcomes can benefit critically ill patients with cancer and COVID-19, providing better ICU triage and, for patients already in the ICU, providing more appropriate therapeutic planning, prognostication and counseling for patients and their relatives.

In critically ill patients with active cancer admitted to ICU due to COVID-19, the objective of the present study was to evaluate the association between cancer-related characteristics and MV use or in-hospital mortality.

## Methods

Cohort multicenter study included patients from ICUs of four hospitals located in São Paulo, Brazil. The AC Camargo Cancer Center ethics committees approved this study (2521/18L) and waived the need for informed consent.

### Patients

We included consecutive adult patients (≥18 years old) with solid tumors or hematologic malignancies and ICU admission due to COVID-19. We excluded patients with cancer remission ≥5 years; decision to forego life-sustaining therapies prior to ICU admission; and admissions for elective postoperative care. If a patient had multiple ICU admissions, only the first was considered. COVID-19 was confirmed by a positive SARS-Cov-2 real-time reverse transcription polymerase chain reaction in a patient with compatible symptoms or chest computerized tomography findings suggestive of COVID-19.

### Data Collection

Variables were prospectively collected. Each center employed a different data entry form. However, all forms had the same fields and used a standardized definition of the variables.

The following cancer-related characteristics were recorded: 1. Cancer status related to cancer treatment, categorized as newly diagnosed without treatment, partial or complete response, progressive cancer despite treatment; 2. Type of cancer (solid tumor or hematologic malignancy); 3. Metastatic tumor; 4. Recent chemotherapy or immunotherapy, defined as therapy in the last 30 days; 5. Lung as the primary site of the solid tumor (pulmonary metastases from other solid tumor sites were not considered as lung cancer); and 6. Performance status, measured with the Eastern Cooperative Oncology Group (ECOG) score.

Upon ICU admission, patient’s demographic characteristics, Simplified Acute Physiology Score (SAPS 3) (22), ECOG performance status (23); the Sequential Organ Failure Assessment Score (SOFA) (24); Charlson comorbidity index (25), and specific comorbidities, including arterial hypertension, diabetes, chronic pulmonary disease, heart disease, and overweight or obesity were recorded. We also recorded the following symptoms and exams associated with COVID-19: acute (or acute-on-chronic) cough, fever and myalgia, number of lymphocytes in blood, arterial lactate, and serum creatinine, c-reactive protein and D-dimer.

During ICU stay, the need for oxygen therapy (nasal cannula, oronasal or non-rebreathing mask), MV for more than >24 h, noninvasive mechanical ventilation (facial mask noninvasive ventilation or high-flow nasal cannula), vasopressors (any dose of noradrenaline, vasopressin, or adrenaline >1 h), and hemodialysis use were recorded. Finally, the in-hospital mortality was recorded.

### Statistical Analysis

Categorical and continuous data were presented as absolute values (percentages) and median (25–75% interquartile range), respectively. Categorical variables were compared using the Chi-square test or Fisher’s exact test, as appropriate. Continuous variables were compared using the Mann–Whitney test.

Multilevel logistic regression models were used to determine the cancer-related characteristics associated with MV use or in-hospital mortality. The cancer-related characteristics were introduced in the multilevel logistic regression model as first-level variables and hospital introduced as second-level variable (random effect) (26). For each cancer-related characteristic, we used a directed acyclic graph to identify confounders (27). Multicollinearity of the confounders was explored using the variance inflation factors and a value >2.5 defined collinearity. Odds ratios (OR) and 95% confidence intervals (CI) were used to measure the association between each variable and MV use or in-hospital mortality. If no confounder was identified, the model was adjusted for age and sex.

To estimate the causal inference of the cancer-related characteristics on MV use, the regression models for cancer status, type of cancer, and performance status were adjusted for age, sex, Charlson comorbidity index and lung cancer. The regression models for recent use of chemotherapy or immunotherapy, and lung cancer were adjusted for age, sex, and Charlson comorbidities index. Finally, for metastatic tumor, the directed acyclic graph method did not detect any confounder, therefore the regression model was adjusted for age and sex, as prespecified (**Supplementary Figure 1**).

To estimate the association between cancer-related characteristics and in-hospital mortality, the regression models of all cancer-related characteristics were adjusted for age, sex, and Charlson comorbidities index (**Supplementary Figure 2**).

Multicollinearity of the confounders was explored using the variance inflation factors and a value >2.5 defined collinearity.

We presented the unadjusted and adjusted odds ratios with 95% CI for each cancer-related characteristic. As sensitivity analysis, we reproduce the multilevel logistic regression models of the primary analysis, but including SAPS 3 score, as a proxy of the acute illness severity. In an additional sensitivity analysis, we included the number of lymphocytes in blood and c-reactive protein level, as confounders, in the multilevel logistic regression.

There were missing values for arterial lactate (31.4%), D-dimer (36.5%), and C-reactive protein (5.1%) upon ICU admission. These missing values were not imputed.

Statistical analyses were performed by SPSS software (Version 23.0. Armonk, NY: IBM Corp). *P*-values ≤0.05 were considered significant. The directed acyclic graphs were created using the browser-based environment DAGitty (28). We followed the recommendations of the STROBE statement that guides the report of observational studies (29) and the guidance for control of confounding and reporting of results in causal inference studies from editors of respiratory, sleep, and critical care journals (27) (See **Supplementary Material** for further details).

## Results

From February 2020 until November 2020, 274 patients with active cancer and COVID-19 were admitted to the ICU in the participating centers and were included in the study.

The characteristics of the patients upon ICU admission are depicted in **Table 1**. Upon ICU admission, the hospital non-survivors had more organs dysfunction, higher D-dimer and C-reactive protein, and lower performance status compared with hospital survivors. Age, sex, type of cancer, site of the primary tumor, and cancer status were similar between survivors and non-survivors. The prevalence of comorbidities was similar between survivors and non-survivors, except for diabetes. No patient received any dose of COVID-19 vaccine.

**Table 1:** Characteristics upon intensive care unit admission of hospital survivors and non-survivors.

Variable	Survivors (n = 119)	Non-survivors (n = 155)	*P*
**Male**	74 (62.2)	85 (54.8)	0.27
**Age (years)**	66 (58–75)	64 (52–74)	0.18
Comorbidities			
Arterial hypertension	62 (52.1)	70 (45.2)	0.27
Diabetes	42 (35.3)	27 (17.4)	<0.01
COPD	15 (12.6)	16 (10.3)	0.57
Heart disease	18 (15.1)	16 (10.3)	0.27
BMI >25 kg/m^2^	60 (50.4)	66 (42.6)	0.22
Chronic kidney failure	12 (10.1)	12 (7.7)	0.52
**Charlson comorbidities index**	6 (3–7)	6 (4–8)	0.15
**SAPS 3**	58 (49–64)	71 (59–82)	<0.01
**SOFA**	1 (0–3)	4 (2–7)	<0.01
	***COVID-19 characteristics***		
**COVID-19 symptoms**			
Fever	68 (57.1)	68 (43.9)	0.03
Acute cough	71 (59.7)	79 (51.0)	0.14
Breathless	76 (63.9)	118 (76.1)	0.03
Myalgia	20 (16.8)	17 (11.0)	0.21
**Laboratory results**			
Arterial lactate (mg/dl) (n = 188)	14 (10–19)	16 (12–23)	0.02
D-dimer (ng/ml) (n = 174)	1,158 (690–1,949)	2,355 (1,093–6,337)	<0.01
Lymphocyte (cells per mm^3^)	720 (450–1,180)	585 (302–1,032)	0.05
C-reactive protein (mg/l) (n = 260)	10 (5–26)	18 (12–27)	<0.01
Creatinine (mg/dl)	0.96 (0.70–1.36)	1.14 (0.71–1.78)	0.04
	***Cancer-related characteristics***		
**Type of cancer**			0.88
Hematologic malignancy	27 (22.7)	33 (20.6)	
Solid tumor	92 (77.3)	122 (78.7)	
**Metastatic tumor**	36 (30.3)	69 (44.5)	0.01
**Type of hematologic malignancy**			0.99
Low-grade hematologic malignancy	18 (15.1)	22 (14.2)	
High-grade hematologic malignancy	9 (7.6)	11 (7.1)	
**Site of solid tumor**			0.26
Prostate	21 (17.6)	14 (9.0)	
Breast	16 (13.4)	25 (16.1)	
Lung	6 (5.0)	14 (9.0)	
Head and neck	6 (5,0)	6 (3.9)	
Colon	7 (5.9)	12 (7.7)	
Bladder	3 (2.5)	7 (4.5)	
Melanoma	6 (5.0)	3 (2.0)	
Others	27 (22.7)	42 (27.1)	
**Lung cancer**	6 (5.0)	14 (9.0)	0.25
**Cancer status**			0.09
Newly diagnosed without treatment	8 (6.7)	18 (11.6)	
Partial or complete response	78 (65.5)	82 (52.9)	
Progressive	33 (27.7)	55 (35.5)	
**Previous cancer treatment**			
Surgery	67 (56.3)	74 (47.7)	0.14
Chemotherapy	71 (59.7)	107 (69.0)	0.08
Recent chemotherapy (<30 days)	23 (19.3)	44 (28.4)	0.12
Immunotherapy	12 (10.1)	15 (9.7)	0.99
Recent Immunotherapy (<30 days)	7 (5.9)	8 (5.2)	0.79
Radiotherapy	36 (30.3)	49 (31.6)	0.99
***Performance status*** **(ECOG)**			<0.01
0–1	95 (79.8)	86 (55.5)	
2	10 (8.4)	35 (22.6)	
3–4	13 (10.8)	34 (21.9)	

COPD, chronic obstructive pulmonary diseases; Heart disease, chronic arrythmia needing treatment, systolic or diastolic heart failure; BMI, body mass index; SAPS 3, simplified acute physiology score 3; SOFA, sequential organ failure assessment score; High-grade hematologic malignancy, Diffuse large B cell non-Hodgkin lymphoma, grade 3 poor differentiated follicular lymphoma, mantle cell lymphoma, peripheral T-cell lymphoma, anaplastic large cell lymphoma, Burkitt lymphoma, acute lymphocytic and myelogenous leukemia. All other hematologic malignancies were considered as low-grade; ECOG, Eastern Cooperative Oncology Group.

Categorical and continuous data were presented as absolute values (percentages) and median (25–75% interquartile range), respectively. Categorical variables were compared using the Chi-square test or Fisher’s exact test, as appropriate. Continuous variables were compared using the Mann–Whitney test.

During ICU stay, hospital non-survivors required more MV, vasopressors, and hemodialysis. The use of oxygen therapy, facial mask noninvasive mechanical ventilation, and high-flow nasal cannula were similar between survivors and non-survivors (**Table 2**).

**Table 2:** Characteristics during ICU stay of hospital survivors and non-survivors.

Variable	Survivors (n = 119)	Non-survivors (n = 155)	*P*
**Oxygen therapy**	112 (94.1)	149 (96.1)	0.77
**High-flow nasal cannula**	15 (12.6)	18 (11.6)	0.85
**Facial mask noninvasive MV**	35 (29.4)	43 (27.7)	0.99
**Invasive MV**	48 (40.3)	128 (82.6)	<0.01
**Vasopressors**	50 (42.0)	130 (83.9)	<0.01
**Hemodialysis**	14 (11.8)	56 (36.1)	<0.01
**Invasive MV duration (days)**	8 (5–12)	10 (6–16)	0.11
**Ventilator-free (days)**	6.0 (3.0–9.3)	1.0 (0.0–3.0)	<0.01
**ICU length of stay (days)**	5 (3–14)	9 (4–17)	0.02
**Hospital length of stay (days)**	22 (13–33)	16 (9–24)	<0.01

MV, mechanical ventilation; Vasopressors, noradrenaline, vasopressin, or adrenaline >1 h.

Categorical and continuous data were presented as absolute values (percentages) and median (25–75% interquartile range), respectively. Ventilator-free days defined as the number of days alive and free from mechanical ventilation for at least 48 consecutive hours.

Categorical variables were compared using the Chi-square test or Fisher’s exact test, as appropriate. Continuous variables were compared using the Mann–Whitney t-test.

### Association With Invasive Mechanical Ventilation

During hospital stay, 176 patients required MV (64.2%) with a mean duration of 9 days (6–15). Fifty patients (28.4%) initiated the MV while in ward or emergency room. In-hospital mortality was 27.4% (18.6–36.2%, 95% CI) for patients who did not require MV, and 72.7% (66.1–79.3%, 95% CI) for patients that required MV.

**Figure 1:**
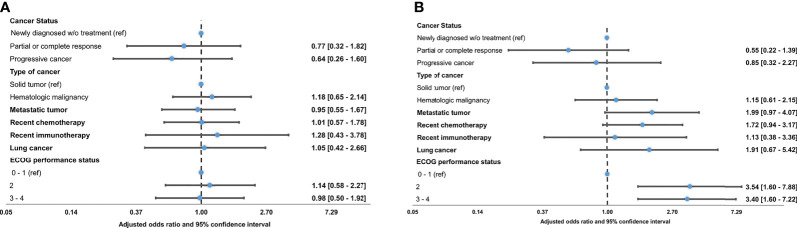
Forest plot of cancer-related characteristics associated with invasive mechanical ventilation use **(A)** or in-hospital mortality **(B)**. Data are adjusted odds ratios with 95% confidence intervals. ECOG, Eastern Cooperative Oncology Group. Panel **(A)** (invasive mechanical ventilation use): Cancer status, type of cancer, and performance status were adjusted for age, sex, Charlson comorbidity index and lung cancer. Recent use of chemotherapy or immunotherapy, and lung cancer were adjusted for age, sex and Charlson comorbidities index. Metastatic tumor was adjusted for age and sex. Panel **(B)** (in-hospital mortality): All models adjusted for age, sex, and Charlson comorbidities index.

In a multivariable model adjusting for confounders, none of the cancer-related characteristics were associated with MV use (**Figure 1A**). The unadjusted models (**Supplementary Figure 3**) and the sensitivity analyses (**Supplementary Figures 4**, **5**) also showed that cancer-related characteristics were not associated with MV.

### Association With In-Hospital Mortality

Approximately 134 patients died in ICU (48.9%, 43.0–54.8%, 95% CI). Among the 140 patients discharged alive from ICU, 21 died in hospital, resulting an in-hospital mortality of 56.6% (50.7–62.5%, 95% CI).

In the adjusted models, a poor performance status (ECOG ≥2) was associated with increased in-hospital mortality (**Figure 1B**). Metastatic tumor was associated with a twofold increase in the in-hospital mortality, but this estimate was imprecise [OR =1.99 (0.97–4.07, 95% CI)]. Likewise, recent chemotherapy was associated with a 1.7-fold increase in mortality, but this estimate was imprecise [OR =1.72 (0.94–3.17, 95% CI)]. Cancer status, cancer type, lung cancer, and recent immunotherapy were not associated with in-hospital mortality. The unadjusted models (**Supplementary Figure 6**) and the sensitivity analyses (**Supplementary Figures 7**, **8**) showed results similar to the primary analysis.

## Discussion

We showed that cancer-related characteristics were not associated with MV use in critically ill patients with COVID-19. We also showed that poor performance status was associated with in-hospital mortality.

The association of cancer-related characteristics with worse outcomes in patients with COVID-19 is controversial, probably because cancer and COVID-19 are diseases with large heterogeneity, that combined with methodological differences turned the studies barely comparable. Moreover, the studies evaluated different populations. Considering the age, the studies included adults, children or a mixed sample of adult and pediatric patients (18, 30). Considering the type of tumor, studies included patients with solid tumors (7), specific solid tumors (lung or thoracic) (19, 20), hematologic malignancies (8), or patients with solid tumors and hematological malignancies with different proportions (12, 18). Considering the COVID-19 aspects, studies included outpatients and inpatients with different levels of COVID-19 severity, including asymptomatic patients (20), patients that went to hospital for scheduled cancer treatment (15), and hospitalized patients (8). The primary outcomes also varied among the studies (8, 15, 17, 20) and the same outcome, such as mortality, had different definitions (8, 9, 13, 18, 30). Regional care differences and SARS-CoV-2 variants may also have contributed to the differences. Nationwide studies showed in-hospital mortality varying from 17% in Germany (31) to 38% in Brazil (32), probably reflecting differences in healthcare system, adherence to best practices, and temporal spread of the pandemic. Some studies were performed in United Kingdom (9) or Brazil (16), where the British Alpha (former B.1.1.7) (33, 34) and the Brazilian Gamma (former P.1) (35) variants are predominant, more transmissible and have an unknown impact on outcomes (33, 34). Finally, the studies employed different methodologies and most included pre-established or *P*-based select confounders, what is not in accordance with the best practices to control of confounding and reporting of results in causal inference studies (27).

It is known that patients with cancer are more susceptible to COVID-19 (1, 2) and have worse outcomes, such as hospitalization and ICU admission (1–5). However, for patients admitted with severe COVID-19, cancer-related characteristics may lose or decrease their impact on need for MV and mortality, as we showed in the present study. A similar finding was reported by Brar et al. in a study that compared hospitalized COVID-19–infected patients without cancer with a matched for cohort of patients with COVID-19 and cancer, showing that cancer was not associated with need for MV or mortality (36).

In the present study, cancer-related characteristics were not associate with MV use. Limited mechanistic data are available on the causes of COVID-19 progression to acute respiratory distress syndrome (ARDS). We know that SARS-CoV-2 has specific virulence mechanisms that, combined with personal innate antiviral response, account for the heterogeneous clinical evolution of patients with COVID-19 (37, 38). This combination, and not the presence of cancer, may be the determinants of progression to moderate or severe ARDS. Corroborating this hypothesis, Xu et al., using a machine learning model, showed that malignancy was not associated with progression to ARDS in patients with COVID-19 (39).

In the present study, no cancer-related characteristic, except performance status, was associated with in-hospital mortality. One could expect that hematologic malignancies (type of cancer) would be associated with higher in-hospital mortality because critically ill patients with hematologic malignancies have presented higher short-term mortality compared with patients with solid tumors (40). However, our results did not show an association of hematologic malignancies with in-hospital mortality. The first hypothesis for this discrepancy is that, in previous studies (40), patients with hematologic malignancies were admitted to ICU for cancer or treatment-related complications, while during the pandemic these patients were admitted for an acute respiratory viral disease. The second hypothesis is that, in the last years, patients with hematologic malignancies had higher reductions in short-term mortality than patients with solid tumors (41). Finally, a study that evaluated in-hospital mortality of patients with cancer admitted to ICU requiring ventilatory support showed that hematologic malignancies were not associated with mortality (42). We also could expect that recent chemotherapy and immunotherapy could impact on in-hospital mortality. However, our results did not show an association of recent cancer treatment with in-hospital mortality. Recent chemotherapy weakens the immune system, while recent immunotherapy enhances it. A weakened immune system could negatively impact on outcomes increasing viral replication but could positively impact avoiding the cytokine storm. On the other hand, an enhanced immune system could positively impact on outcomes decreasing viral replication, but negatively promoting the cytokine storm. Therefore, the impact of recent chemotherapy and immunotherapy can vary unexpected and in opposite direction.

We showed that poor performance status was associated with in-hospital mortality. Poor performance status has been associated with in-hospital mortality in patients with cancer and acute respiratory failure due to COVID-19 (14) or other causes (42). Zampieri et al. showed that poor performance status was associated with increased in-hospital mortality of critically ill patients, regardless of the cancer-related characteristics (43). Two studies did not show the association of poor performance status with worse outcomes in patients with cancer and COVID-19 (12, 20). However, both studies mixed outpatients with inpatients, with COVID-19 severity ranging from asymptomatic to critically-ill patients, and one study evaluated only patients with thoracic tumors (20).

In critically ill patients with COVID-19, the knowledge of cancer-related characteristics associated with worse outcomes can be used to prompt a closer monitoring, avoiding ICU admission delay, which has been associated with higher mortality in patients with cancer and acute respiratory failure (44). Additionally, in patients with progressive cancer despite treatment, the knowledge can allow better ICU triage, following the principles of beneficence, non-maleficence, and autonomy (45). Information about the cancer-related characteristics associated with worse outcomes are important for providing proper prognostication, therapeutic planning and counseling for all patients and their relatives. Finally, our findings suggest that cancer-related characteristics should not motivate intensive care unit triage and treatment decisions, except when performance status is poor.

Our study has limitations inherent to observational studies. The care of patients across different centers might not be comparable. However, we employed a statistical analysis designed to deal with heterogeneity among centers (26), and the heterogeneous care reflects real-world conditions. Although consecutive inclusion mitigates the risk of selection bias, we excluded critically ill patients with cancer and COVID-19 that were not admitted to the ICU due to perceived futility of ICU admission and it is possible that those patients had more metastatic disease, progressive cancer and poor performance status. In addition, our sample size may have been insufficient to detect the association between cancer-related characteristics, such as metastatic cancer and recent chemotherapy with mortality.

## Conclusions

In patients with active cancer admitted to ICU due to COVID-19, a poor performance status is associated with increased in-hospital mortality. Metastatic tumor and recent chemotherapy are associated with approximately twofold increase in hospital mortality, but the estimates are imprecise. The cancer status, type of cancer, lung cancer, and recent immunotherapy are not associated with increased in-hospital mortality. Cancer-related characteristics are not associated with increased invasive mechanical ventilation use. The results, however, should be cautiously interpreted due to the observational design of the study.

## Data Availability Statement

The raw data supporting the conclusions of this article will be made available by the authors, without undue reservation.

## Ethics Statement

The AC Camargo Cancer Center ethics committees approved this study (2521/18L) and waived the need for informed consent. Written informed consent for participation was not required for this study in accordance with the national legislation and the institutional requirements.

## Author Contributions

PC, RT, and AP: Substantial contributions to the conception of the work, acquisition, and interpretation of data. Revised the manuscript critically for important intellectual content. IF, VO, PS, RC, AK, RF, WP, EC, TM, FC, and JF: Acquisition and interpretation of data and revised the manuscript critically for important intellectual content. All authors contributed to the article and approved the submitted version.

## Funding

This study was funded by the intensive care unit of the AC Camargo Cancer Center.

## Conflict of Interest

The authors declare that the research was conducted in the absence of any commercial or financial relationships that could be construed as a potential conflict of interest.

## Publisher’s Note

All claims expressed in this article are solely those of the authors and do not necessarily represent those of their affiliated organizations, or those of the publisher, the editors and the reviewers. Any product that may be evaluated in this article, or claim that may be made by its manufacturer, is not guaranteed or endorsed by the publisher.
