# Supplementary material for: Cancer-Related Characteristics Associated With Invasive Mechanical Ventilation or In-Hospital Mortality in Patients With COVID-19 Admitted to ICU: A Cohort Multicenter Study
Source: Front Oncol. 2021 Nov 30;11:746431. doi: 10.3389/fonc.2021.746431 (PMC8668608; doi:10.3389/fonc.2021.746431)
Supplement: Supplementary file 1 [file DataSheet_1.pdf]

Supplementary Material

Cancer-related characteristics associated with invasive mechanical ventilation or in-hospital mortality in patients with COVID-19 admitted to ICU: a cohort multicenter study

Pedro Caruso[1,2], Renato Scarsi Testa[1], Isabel Cristina Lima Freitas[1], Ana Paula Agnolon Praça[1], Valdelis Novis Okamoto[1], Pauliane Vieira Santana[1], Ramon Teixeira Costa[1], Alexandre de Mello Kawassaki[1,2], Renata Rego Lins Fumis[3], Wilber Antonio Pino Illanes[3], Eduardo Leite Vieira Costa[2,3], Thais Dias Midega[4], Thiago Domingos Correa[4], Ph.D (thiago.correa@einstein.br), Fabricio Rodrigo Torres de Carvalho[1,4], Juliana Carvalho Ferreira[1,2].

[1] Intensive Care Unit – AC Camargo Cancer Center, Sao Paulo - Brazil.

[2] Divisao de Pneumologia, Instituto do Coracao (InCor), Hospital das Clinicas HCFMUSP, Faculdade de Medicina, Universidade de Sao Paulo, Sao Paulo - Brazil.

[3] Hospital Sírio-Libanês, Research and Education Institute, Sao Paulo – Brazil.

[4] Department of Critical Care Medicine, Hospital Israelita Albert Einstein, Sao Paulo – Brazil.

Supplemental Table 1. Overview of the studies that evaluated the association of cancer-related characteristics with worse outcomes in patients with COVID-19.

Cancer-related characteristics	Associated with worse outcomes	NOT associated with worse outcomes
**Recent chemotherapy**	**Larfors [1] - retrospective multicenter**<br>- Outcomes: ICU admission, 30d mortality<br>- Chemotherapy < 3 months<br>- Population: in and outpatient - solid and hematologic<br>**Zhang [2] - retrospective multicenter**<br>Outcomes: Composite (ICU admission, MV or death)<br>- Chemotherapy < 14 days<br>- Population: hospitalized - solid tumors<br>**Cattaneo [3] - retrospective two centers**<br>- Outcome: 30d mortality<br>- Chemotherapy < one month<br>- Population: hospitalized - hematologic malignancies<br>**Lee [4] - observational prospective multicenter**<br>- Outcome: in-hospital mortality<br>- Chemotherapy < 28 days<br>- Population: in and outpatient - solid and hematologic<br>- Association only with hematologic malignancies<br>**Grivas [5] - observational prospective multicenter**<br>- Outcomes: composite (ICU, MV and 30d mortality)<br>- Chemotherapy < 3 months<br>- Population: in and outpatient - solid and hematologic	**Luo [6] - retrospective single-center**<br>- Outcomes: ICU admission, MV, death<br>- Chemotherapy < 3 weeks<br>- Population: in and outpatients – lung tumors<br>**Robilotti [7] – retrospective single-center**<br>- Outcomes: hospitalization, MV, high-flow oxygen therapy<br>- Chemotherapy < 30 days.<br>- Population: in and outpatient – solid and hematologic<br>**Jee [8]- retrospective single-center**<br>- Outcomes: Composite (ICU admission, death)<br>- Chemotherapy < 35 days<br>- Population: in and outpatient - solid and hematologic<br>**Garassino [9] – observational cross-sectional and longitudinal multicenter**<br>- Outcomes: hospitalization, death<br>- Chemotherapy < 3 months.<br>- Population: asymptomatic and symptomatic in and outpatient - thoracic tumor<br>**Mehta [10]- retrospective single center**<br>- Outcomes: in-hospital mortality<br>- Undefined active chemotherapy<br>- Population: adult and pediatric hospitalized - solid tumor and hematologic malignancies<br>**Yarza [11]- observational prospective single-center**<br>- Outcomes: respiratory failure (ARDS and non-ARDS)<br>- Chemotherapy < 4 weeks<br>- Population: in and outpatients – solid tumors<br>**Liévre [12]– retrospective multicenter**<br>-Outcome: ICU admission, MV, death<br>- Chemotherapy < 3 months<br>- Population: in and outpatient – solid tumors

**Recent immunotherapy**	**Robilotti [7] – retrospective single-center**<br>- Outcomes: hospitalization, MV, high-flow oxygen therapy<br>- Immunotherapy < 30 days.<br>- Population: in and outpatient - solid and hematologic	**Luo [6] – retrospective single-center**<br>- Outcomes: ICU admission, MV, death<br>- Immunotherapy < 3 weeks<br>- Population: in and outpatient - lung tumors<br>**Grivas [5] - observational prospective multicenter**<br>- Outcomes: composite (ICU, MV and 30d mortality)<br>- Immunotherapy < 3 months<br>- Population: in and outpatient - solid and hematologic
**Type of cancer (Hematologic or solid)**	**Lee [4] - observational prospective multicenter**<br>- Outcome: in-hospital mortality<br>- Population: in and outpatient - solid and hematologic<br>- Association only with leukemia.<br>**Grivas [5] - observational prospective multicenter**<br>- Outcomes: composite (ICU, MV and 30d mortality)<br>- Population: in and outpatient – solid and hematologic<br>**Larfors [1] - retrospective multicenter**<br>- Outcomes: ICU admission, 30d mortality<br>- Population: in and outpatient - solid and hematologic<br>**Dai [13] - retrospective multicenter**<br>- Outcomes: composite (severe symptoms, ICU, MV, death)<br>- Population: hospitalized - solid and hematologic<br>**Robilotti [7] - retrospective single-center**<br>- Outcomes: hospitalization, MV, high-flow oxygen therapy<br>- Population: in and outpatient - solid and hematologic<br>**Jee [8] - retrospective single-center**<br>- Outcomes: Composite (ICU admission, death)<br>- Population: in and outpatient - solid and hematologic	**Shoumariyeh [14] - retrospective single-center**<br>- Outcomes: composite (oxygen support, time to transfer to ICU, time to clinical improvement and overall survival, duration of hospitalization and time to severe events).<br>- Population: hospitalized - solid and hematologic<br>**Russel [15] - observational prospective single-center**<br>- Outcome: COVID-19 severity and death.<br>- Population: hospitalized or scheduled to cancer treatment - solid and hematologic
Performance status - ECOG"	**Kuderer [16] - observational prospective multicenter**<br>- Outcome: 30d mortality<br>- Population: in and outpatient - solid and hematologic	**Jee [8] - retrospective single-center**<br>- Outcomes: Composite (ICU admission, death)<br>- Population: in and outpatient - solid and hematologic

	- ECOG >2 associated with mortality.<br>**Grivas [5] - observational prospective multicenter**<br>- Outcomes: composite (ICU, MV and 30d mortality)<br>- Population: in and outpatient - solid and hematologic<br>- ECOG ≥ 1 associated with severity and mortality<br>**Liévre [12]– retrospective multicenter**<br>-Outcome: ICU admission, MV, death<br>- Population: in and outpatient – solid tumors<br>- ECOG >2 associated with severity and mortality	**Garassino [9] - observational cross-sectional and longitudinal multicenter**<br>- Outcomes: hospitalization, death<br>- Population: asymptomatic and symptomatic in and outpatient - thoracic tumor
**Lung cancer**	**Yang [17] - meta-analysis**<br>-Five studies included<br>**Larfors [1] – retrospective multicenter**<br>- Outcomes: ICU admission, 30d mortality<br>- Population: in and outpatient - solid and hematologic<br>**Jee [8]- retrospective single center**<br>- Outcomes: Composite (ICU admission, death)<br>- Population: in and outpatient - solid and hematologic	**Dai [13] – retrospective multicenter**<br>- Outcomes: composite (severe symptoms, ICU, MV, death)<br>- Population: hospitalized - solid and hematologic<br>**Ferrari [18]– longitudinal multicenter**<br>- Outcomes: all-cause mortality<br>- Population: in and outpatients - solid and hematologic<br>**Yarza [11]- observational prospective single-center**<br>- Outcomes: respiratory failure (ARDS and non-ARDS)<br>- Population: in and outpatient - solid tumor<br>**Liévre [12]– retrospective multicenter**<br>-Outcome: ICU admission, MV, death<br>- Population: in and outpatient – solid tumors
**Cancer status**	**Pinaña [19] – retrospective multicenter**<br>- Outcomes: severe COVID-19 and 45d mortality<br>- Cancer status: complete/partial remission, uncontrollable, active not requiring therapy<br>- Population: pediatric and adult - hematologic<br>**Russel [15] - observational prospective single-center**<br>- Outcome: COVID-19 severity and death<br>- Cancer status: No active, curative, palliative treatment	**Cattaneo [3] – retrospective two centers**<br>- Outcome: 30d mortality<br>- Cancer status: Remission, stable, relapse/refratory<br>- Population: hospitalized hematologic malignancies<br>**Jee [8] – retrospective single-center**<br>- Outcomes: Composite (ICU admission, death)<br>- Cancer status: active or inactive<br>- Population: in and outpatient - solid and hematologic<br>**Luo [6] – retrospective single center**<br>- Outcomes: ICU admission, MV, death

	- Population: hospitalized or scheduled to cancer treatment - solid and hematologic<br>**Kuderer [16] - observational prospective multicenter**<br>- Outcome: 30d mortality<br>- Cancer status: remission, stable, progressive<br>- Population: in and outpatient - solid and hematologic<br>**Grivas [5] - observational prospective multicenter**<br>- Outcomes: composite (ICU, MV and 30d mortality)<br>- Cancer status: responding, stable, progressing<br>- Population: in and outpatient - solid and hematologic<br>**Ferrari [18] - longitudinal multicenter**<br>- Outcomes: all-cause mortality<br>- Cancer status: curative or noncurative<br>- Population: in and outpatient - solid and hematologic	- Cancer status: active or inactive cancer treatment<br>- Population: in and outpatient - lung tumor
**Metastatic tumor**	**Dai [13] – retrospective multicenter**<br>- Outcomes: composite (severe symptoms, ICU, MV, death)<br>- Population: hospitalized - solid and hematologic	**Robilotti [7] – retrospective single-center**<br>- Outcomes: hospitalization, MV, high-flow oxygen therapy<br>- Population: in and outpatient - solid and hematologic<br>**Jee [8] – retrospective single-center**<br>- Outcomes: Composite (ICU admission, death)<br>- Population: in and outpatient - solid and hematologic<br>**Luo [6] – retrospective single-center**<br>- Outcomes: ICU admission, MV, death<br>- Population: in and outpatient - lung tumor<br>**Mehta [10]– retrospective single-center**<br>- Outcomes: in-hospital mortality<br>- Population: adult and pediatric hospitalized - solid and hematologic<br>**Liévre [12]– retrospective multicenter**<br>-Outcome: ICU admission, MV, death<br>- Population: in and outpatient – solid tumors

Legends: ICU = Intensive Care Unit; 30d/45d mortality = 30-day or 45-day mortality; MV = mechanical ventilation; ARDS = acute respiratory distress syndrome/ ECOG = Eastern Cooperative Oncology Group.

Methods

We designed an observational cohort multicenter study including patients from ICUs of the following four different hospitals located in São Paulo- Brazil: 1. AC Camargo Cancer Center, a private hospital dedicated to cancer care with 447 beds (50 ICU beds); 2. Hospital das Clínicas da Faculdade de Medicina de São Paulo, a public tertiary teaching hospital with 2,500 beds (300 ICU beds); 3. Hospital Sírio-Libanês, a private tertiary hospital with 466 beds (47 ICU beds); and 4. Hospital Israelita Albert Einstein, a private tertiary teaching hospital with 630 beds (40 ICU beds).

Data collection

Variables were prospectively collected. Each center employed a different data entry form. However, all forms had the same fields and used a standardized definition of the variables. One author (PC) from the coordinating center (AC Camargo Cancer Center) trained the researchers that collected the cancer-related variables in the other centers. All forms were merged into a single SPSS file. In the case any discrepancy was noted, the coordinating center contacted the researcher to solve the discrepancy.

We recorded the following comorbidities: arterial hypertension, diabetes, chronic pulmonary disease (chronic obstructive pulmonary disease or chronic restrictive pulmonary disease), heart diseases (chronic arrythmia needing treatment, systolic or diastolic heart failure), and overweight or obesity (body mass index > 25 kg/m^2). We also recorded the following symptoms and laboratory exams associated with Covid-19: acute (or acute-on-chronic) cough, fever and myalgia, number of lymphocytes per mm^3, creatinine in mg/dl, c-reactive protein in in mg/dl, D-dimer in ng/ml and arterial lactate in mg/dl.

During ICU stay, the need for oxygen therapy (nasal cannula, oronasal or non-rebreathing mask), MV for more than > 24h, noninvasive mechanical ventilation (facial mask noninvasive ventilation or high-flow nasal cannula), vasopressors (any dose of noradrenaline, vasopressin, or adrenaline > 1 hour), and hemodialysis use were recorded.

Statistical analysis

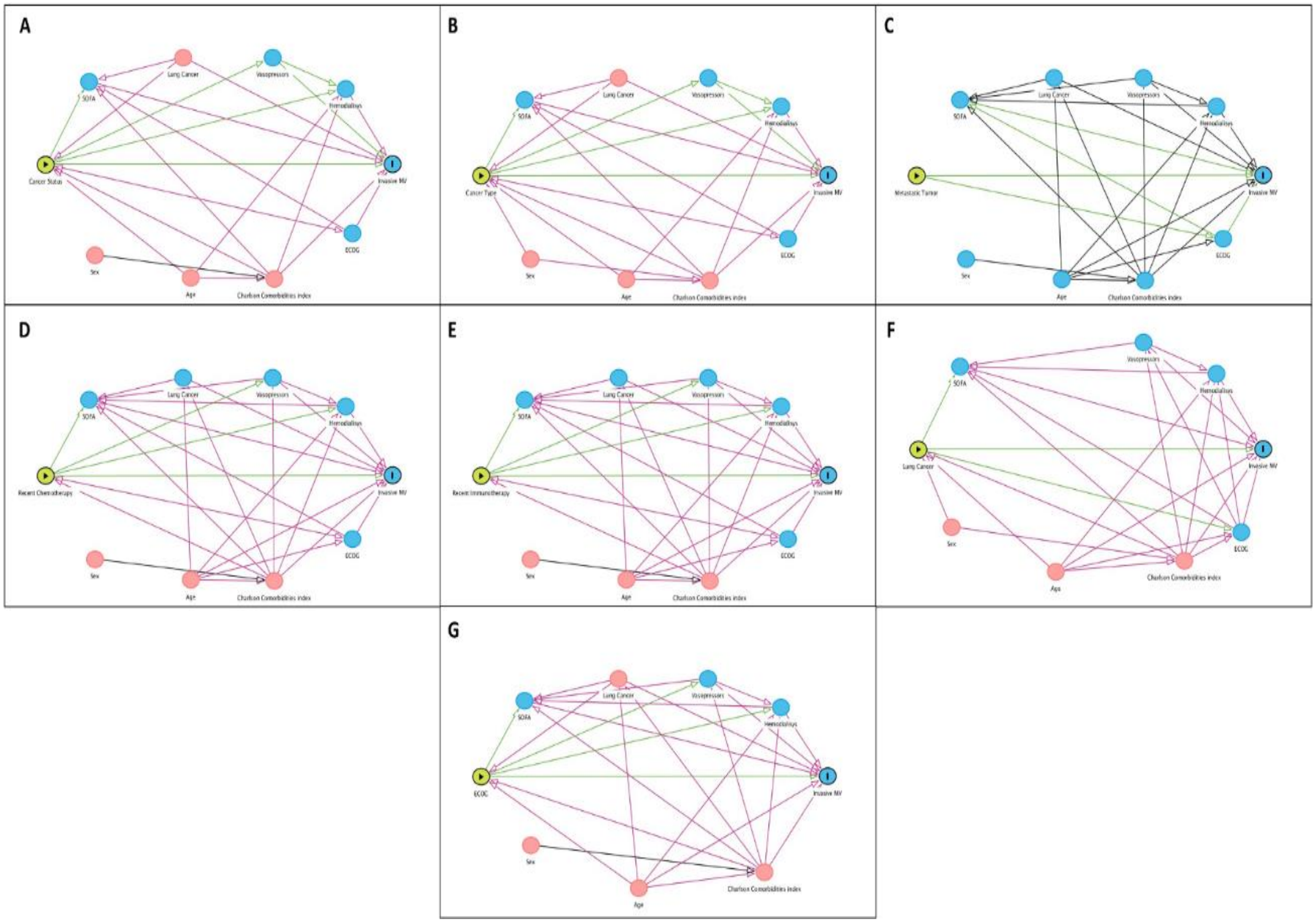

Supplementary Figure 1. Panels A to G depict direct acyclic graphics (DAG) for identification of confounders for the association of each cancer-related characteristic with the need for invasive mechanical ventilation. Panel A = cancer status; Panel B = cancer type; Panel C= metastatic tumor; Panel D = recent chemotherapy; Panel E = recent immunotherapy; Panel F = lung cancer; and Panel G = performance status measured using ECOG. The light red circles were considered confounders. The greenish yellow circles were the evaluated cancer-related characteristic.

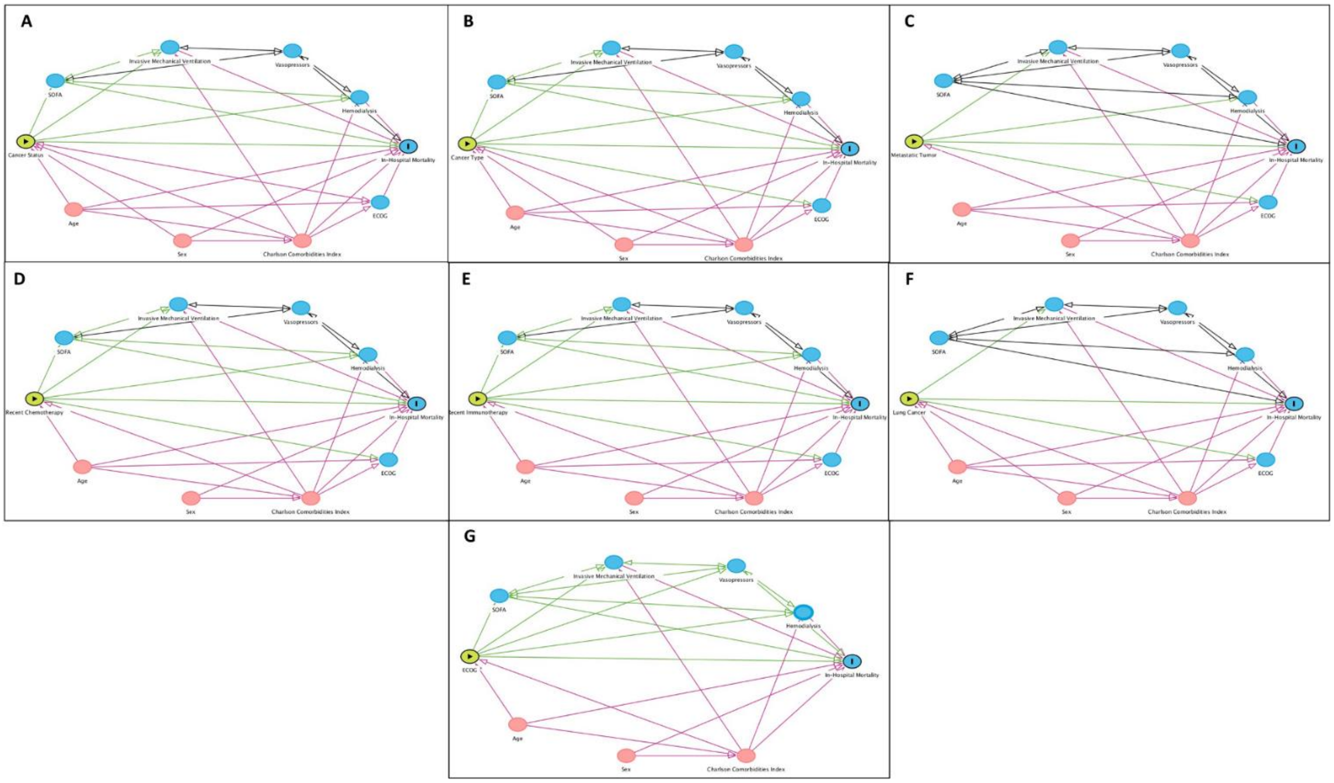

Supplementary Figure 2. Panels A to G depict direct acyclic graphics (DAG) for identification of confounders for the association of each cancer-related characteristic with in-hospital mortality. Panel A = cancer status; Panel B = cancer type; Panel C= metastatic tumor; Panel D = recent chemotherapy; Panel E = recent immunotherapy; Panel F = lung cancer; and Panel G = performance status measured using ECOG. The light red circles were considered confounders. The greenish yellow circles were the evaluated cancer-related characteristic.

Results

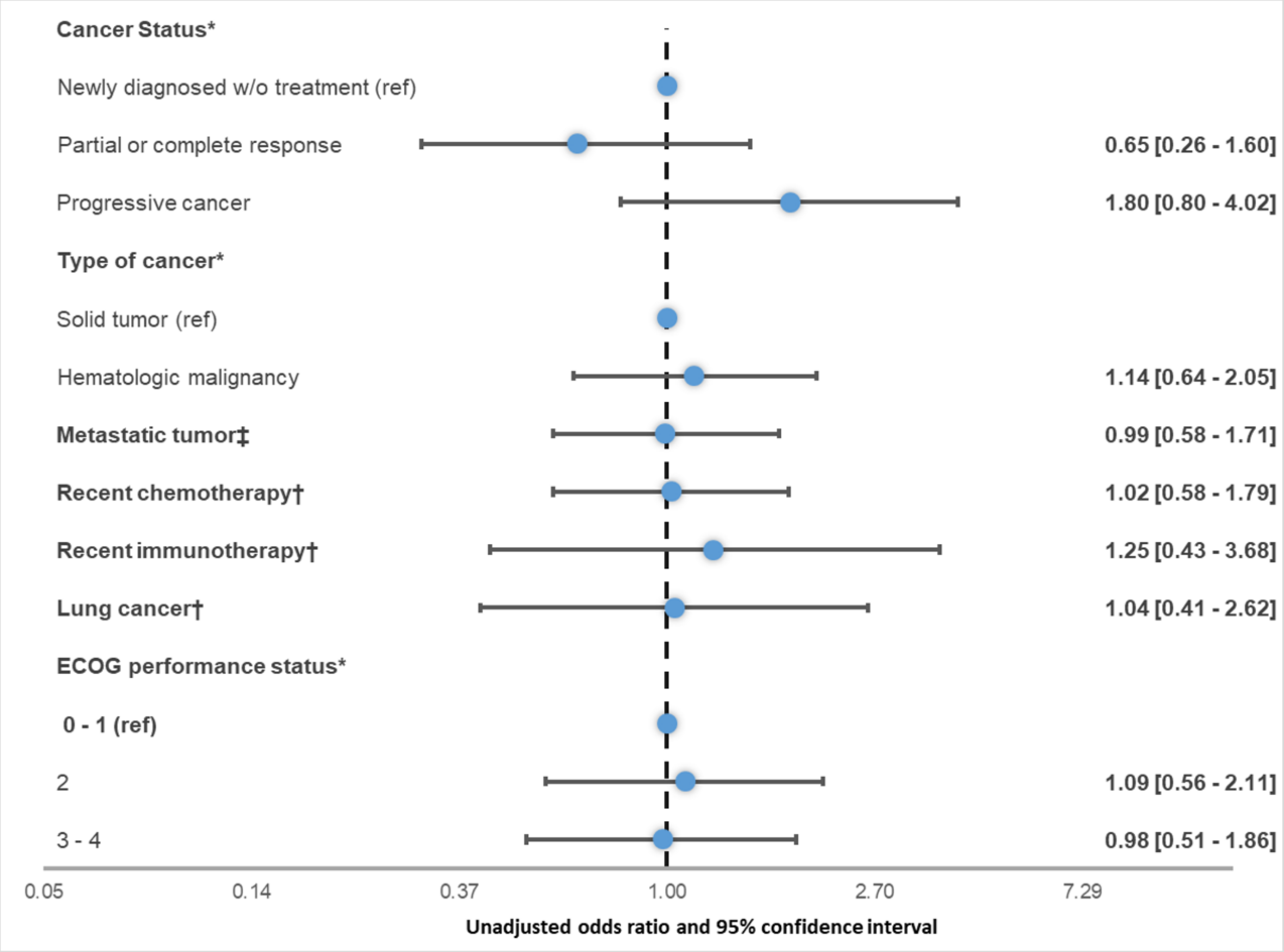

Supplementary Figure 3. Forest plot with unadjusted odds ratio of cancer-related characteristics associated with invasive mechanical ventilation use.

Data are unadjusted odds ratios with 95% confidence intervals. ECOG=Eastern Cooperative Oncology Group.

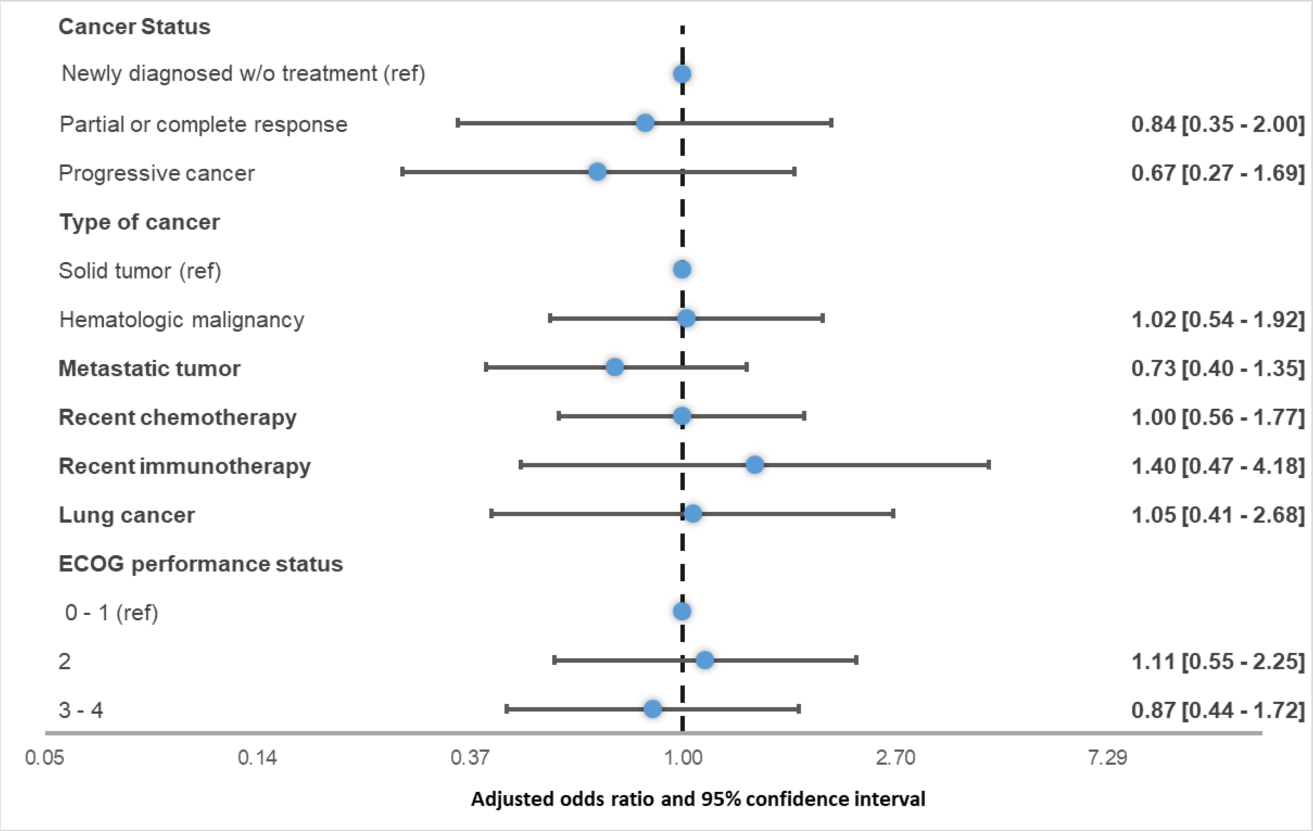

Supplementary Figure 4. Forest plot of cancer-related characteristics associated with invasive mechanical ventilation use, including SAPS 3 as a confounder (sensitivity analysis).

Data are adjusted odds ratios with 95% confidence intervals. ECOG=Eastern Cooperative Oncology Group.

Cancer status, type of cancer, and performance status were adjusted for age, sex, Charlson comorbidity index, lung cancer, and SAPS 3 score. Recent use of chemotherapy or immunotherapy, and lung cancer were adjusted for age, sex, Charlson comorbidities index, and SAPS3 score. Metastatic tumor was adjusted for age, sex, and SAPS 3 score.

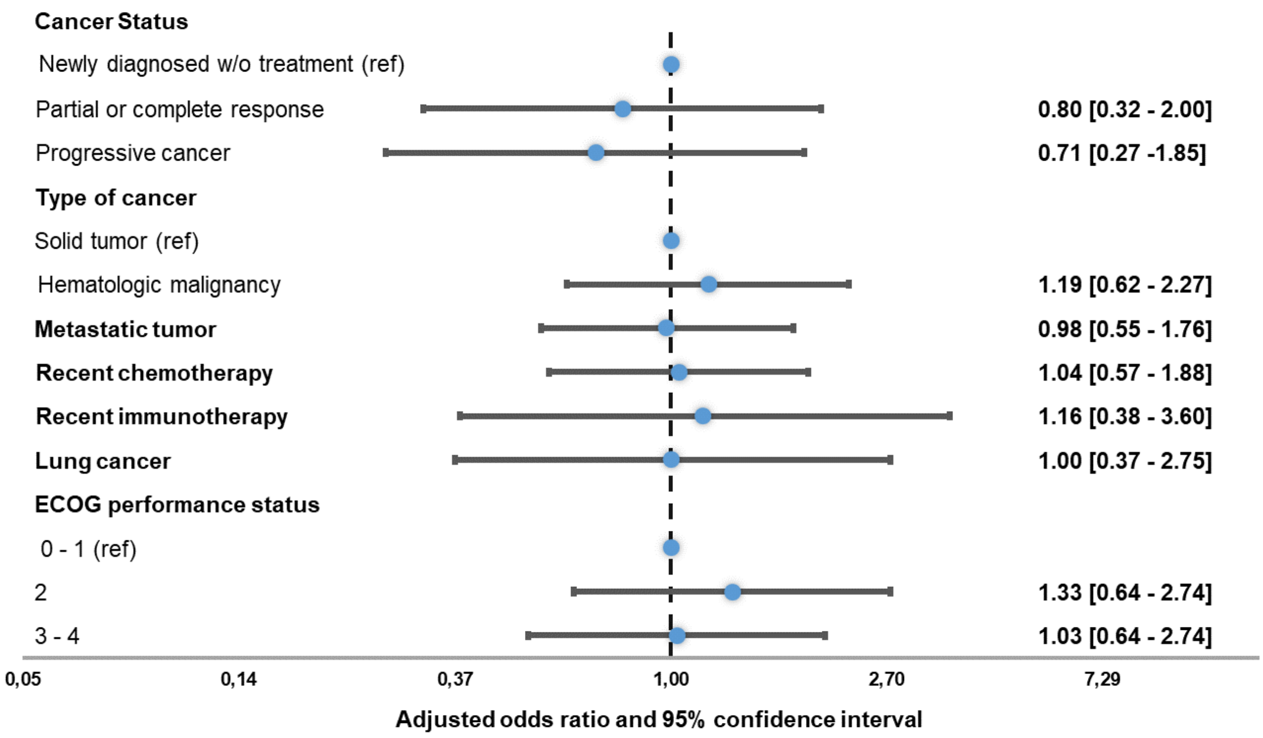

Supplementary Figure 5. Forest plot of cancer-related characteristics associated with invasive mechanical ventilation use including number of lymphocytes in blood and c-reactive protein level as confounders (sensitivity analysis).

Data are adjusted odds ratios with 95% confidence intervals. ECOG=Eastern Cooperative Oncology Group.

Cancer status, type of cancer, and performance status were adjusted for age, sex, Charlson comorbidity index, lung cancer, lymphocytes and c-reactive protein. Recent use of chemotherapy or immunotherapy, and lung cancer were adjusted for age, sex, Charlson comorbidities index, lymphocytes and c-reactive protein. Metastatic tumor was adjusted for age, sex, lymphocytes and c-reactive protein.

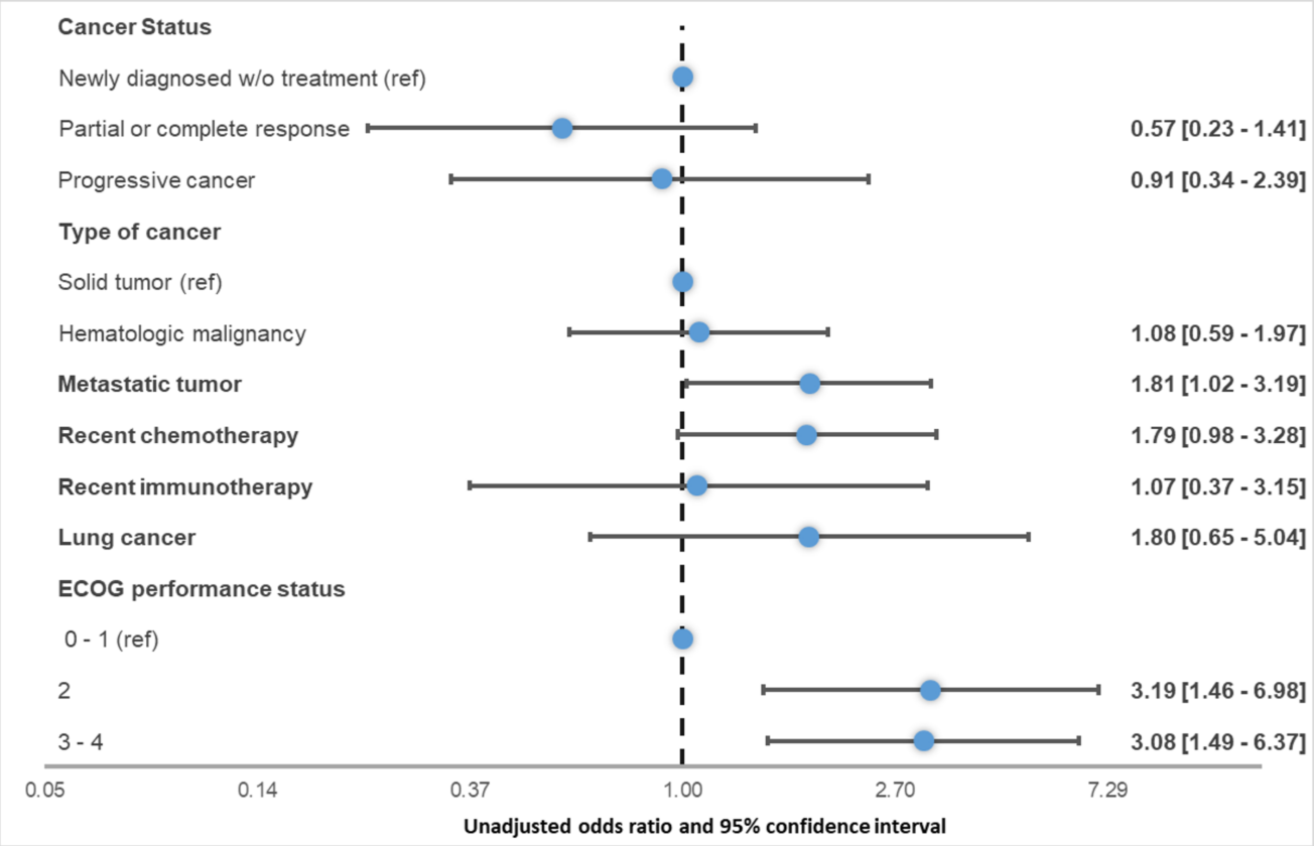

Supplementary Figure 6. Forest plot with unadjusted odds ratios of cancer-related characteristics associated with in-hospital mechanical ventilation.

Data are unadjusted odds ratios with 95% confidence intervals. ECOG=Eastern Cooperative Oncology Group.

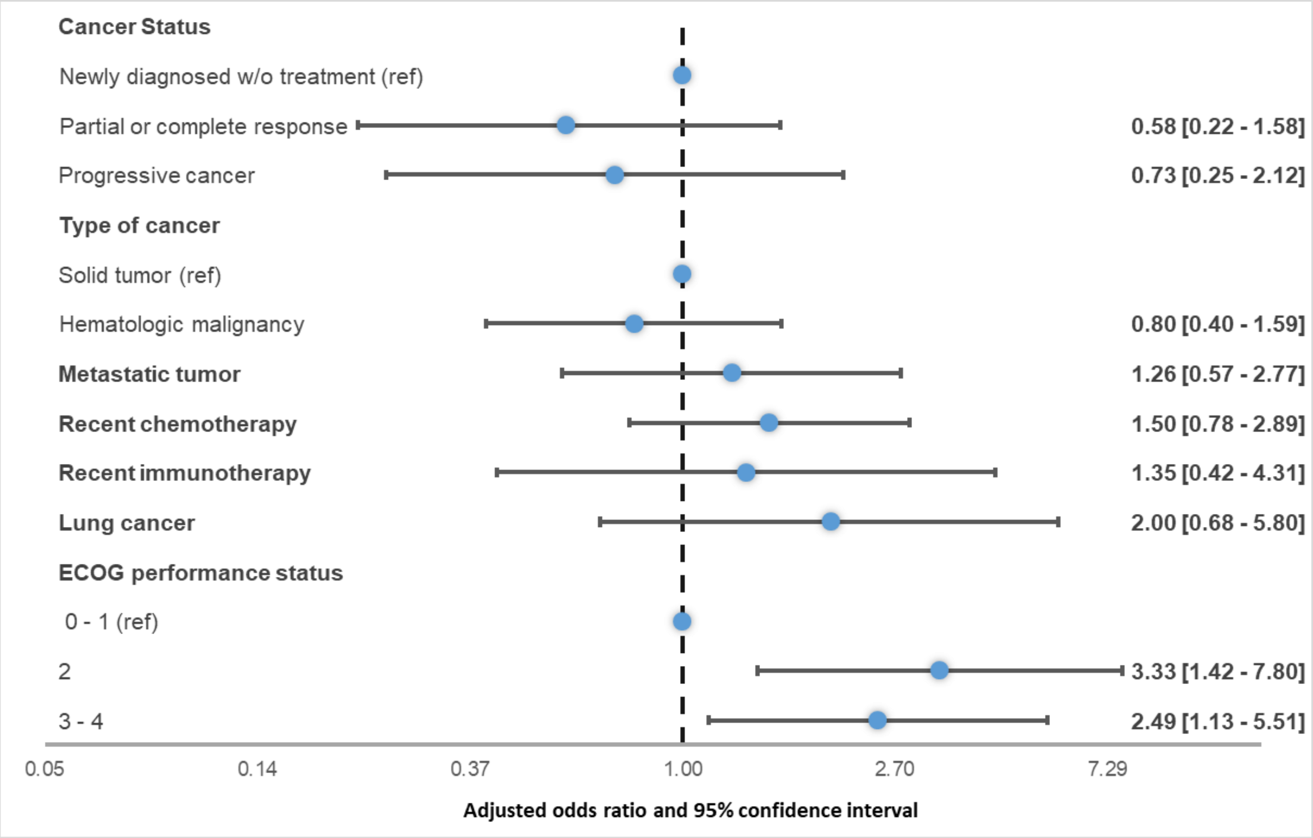

Supplementary Figure 7. Forest plot of cancer-related characteristics associated with in-hospital mortality, including SAPS 3 as a confounder (sensitivity analysis).

Data are adjusted odds ratios with 95% confidence intervals. ECOG=Eastern Cooperative Oncology Group.

All models adjusted for age, sex, Charlson comorbidities index, and SAPS 3 score.

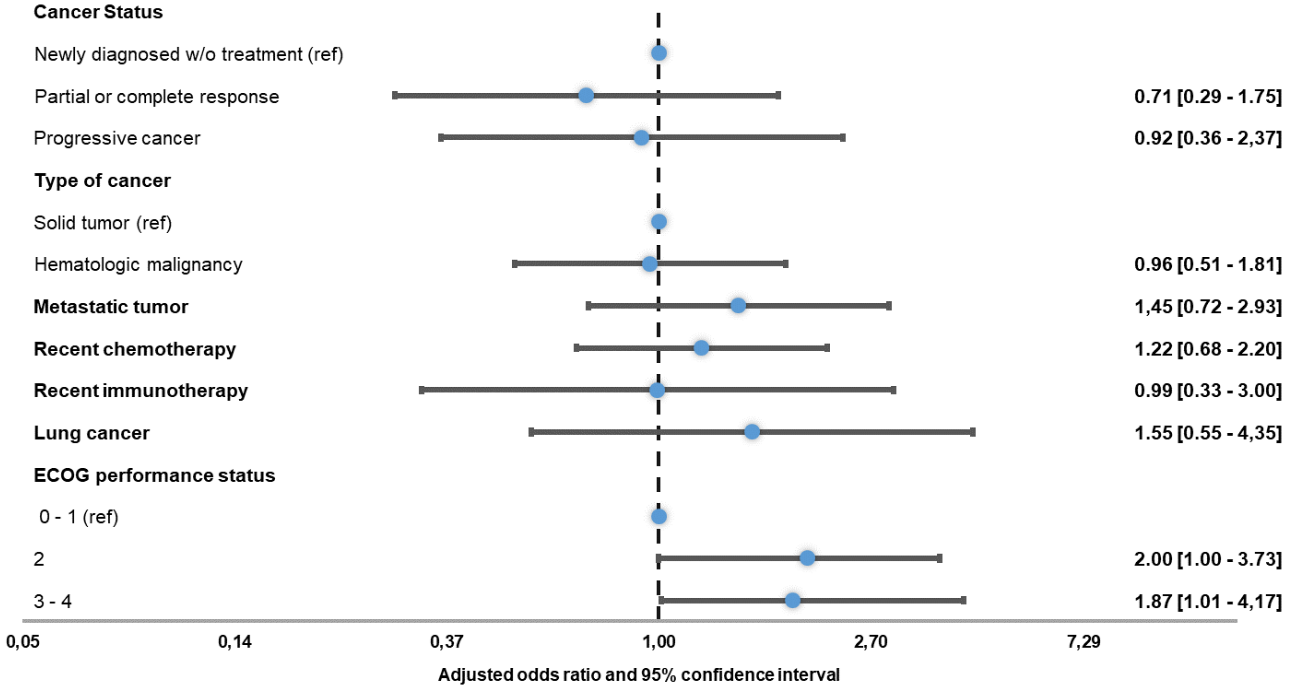

Supplementary Figure 8. Forest plot of cancer-related characteristics associated with in-hospital mortality including number of lymphocytes in blood and c-reactive protein level as confounders (sensitivity analysis).

Data are adjusted odds ratios with 95% confidence intervals. ECOG=Eastern Cooperative Oncology Group.

All models adjusted for age, sex, Charlson comorbidities index, number of lymphocytes in blood, and c-reactive protein level.

References

1. Larfors G, Pahnke S, State M, Fredriksson K, Pettersson D. Covid-19 intensive care admissions and mortality among swedish patients with cancer. Acta Oncol (Madr). 2021;60(1):32-34.
2. Zhang L, Zhu F, Xie L, et al. Clinical characteristics of COVID-19-infected cancer patients: a retrospective case study in three hospitals within Wuhan, China. Ann Oncol. 2020;31(7):894-901.
3. Cattaneo C, Daffini R, Pagani C, et al. Clinical characteristics and risk factors for mortality in hematologic patients affected by COVID-19. Cancer. 2020;126(23):5069-5076.
4. Lee LYW, Cazier JB, Starkey T, et al. COVID-19 prevalence and mortality in patients with cancer and the effect of primary tumour subtype and patient demographics: a prospective cohort study. Lancet Oncol. 2020;21(10):1309-1316.
5. Grivas P, Khaki AR, Wise-Draper TM, et al. Association of Clinical Factors and Recent Anti-Cancer Therapy with COVID-19 Severity among Patients with Cancer: A Report from the COVID-19 and Cancer Consortium. Ann Oncol Off J Eur Soc Med Oncol. 2021;0(0).
6. Luo J, Rizvi H, Preeshagul IR, et al. COVID-19 in patients with lung cancer. Ann Oncol. 2020;31(10):1386-1396.
7. Robilotti E V., Babady NE, Mead PA, et al. Determinants of COVID-19 disease severity in patients with cancer. Nat Med. 2020;26(8):1218-1223.
8. Jee J, Foote MB, Lumish M, et al. Chemotherapy and COVID-19 Outcomes in Patients with Cancer. J Clin Oncol. 2020;38(30):3538-3546.
9. Garassino MC, Whisenant JG, Huang LC, et al. COVID-19 in patients with thoracic malignancies (TERAVOLT): first results of an international, registry-based, cohort study. Lancet Oncol. 2020;21(7):914-922.
10. Mehta V, Goel S, Kabarriti R, et al. Case fatality rate of cancer patients with COVID-19 in a New York Hospital system. Cancer Discov. 2020;10(7):935-941.
11. Yarza R, Bover M, Paredes D, et al. SARS-CoV-2 infection in cancer patients undergoing active treatment: analysis of clinical features and predictive factors for severe respiratory failure and death. Eur J Cancer. 2020;135:242-250.
12. Lièvre A, Turpin A, Ray-Coquard I, et al. Risk factors for Coronavirus Disease 2019 (COVID-19) severity and mortality among solid cancer patients and impact of the disease on anticancer treatment: A French nationwide cohort study (GCO-002 CACOVID-19). Eur J Cancer. 2020;141:62-81.
13. Dai M, Liu D, Liu M, et al. Patients with cancer appear more vulnerable to SARS-CoV-2: A multicenter study during the COVID-19 outbreak. Cancer Discov. 2020;10(6):783.

14. Shoumariyeh K, Biavasco F, Ihorst G, et al. Covid-19 in patients with hematological and solid cancers at a Comprehensive Cancer Center in Germany. Cancer Med. 2020;9(22):8412-8422.

15. Russell B, Moss C, Papa S, et al. Factors Affecting COVID-19 Outcomes in Cancer Patients: A First Report From Guy's Cancer Center in London. Front Oncol. 2020;10.

16. Kuderer NM, Choueiri TK, Shah DP, et al. Clinical impact of COVID-19 on patients with cancer (CCC19): a cohort study. Lancet. 2020;395(10241):1907-1918.

17. Ludi Yang et al. Effects of cancer on patients with COVID-19: a systematic review and meta-analysis of 63,019 participants. Cancer Biol Med. 2021;18(1):298-307.

18. Ferrari BL, Ferreira CG, Menezes M, et al. Determinants of COVID-19 Mortality in Patients with Cancer from a Community Oncology Practice in Brazil. J Glob Oncol. 2021;7(7):46-55.

19. Piñana JL, Martino R, García-García I, et al. Risk factors and outcome of COVID-19 in patients with hematological malignancies. Exp Hematol Oncol. 2020;9(1).
